# Updated Tactile Feedback with a Pin Array Matrix Helps Blind People to Reduce Self-Location Errors

**DOI:** 10.3390/mi9070351

**Published:** 2018-07-14

**Authors:** Luca Brayda, Fabrizio Leo, Caterina Baccelliere, Elisabetta Ferrari, Claudia Vigini

**Affiliations:** 1 Research Unit of Robotics, Brain and Cognitive Sciences, Fondazione Istituto Italiano di Tecnologia, Genoa 16153, Italy; fabrizio.leo@iit.it (F.L.); caterina.baccelliere@gmail.com (C.B.); elisabettaferrari4@gmail.com (E.F.); 2 Istituto David Chiossone Onlus, Genoa 16122, Italy; vigini@chiossone.it

**Keywords:** spatial representation, tactile maps, visual impairment, blindness, pin array matrix, tactile feedback, orientation and mobility, navigation, tactile graphics

## Abstract

Autonomous navigation in novel environments still represents a challenge for people with visual impairment (VI). Pin array matrices (PAM) are an effective way to display spatial information to VI people in educative/rehabilitative contexts, as they provide high flexibility and versatility. Here, we tested the effectiveness of a PAM in VI participants in an orientation and mobility task. They haptically explored a map showing a scaled representation of a real room on the PAM. The map further included a symbol indicating a virtual target position. Then, participants entered the room and attempted to reach the target three times. While a control group only reviewed the same, unchanged map on the PAM between trials, an experimental group also received an updated map representing, in addition, the position they previously reached in the room. The experimental group significantly improved across trials by having both reduced self-location errors and reduced completion time, unlike the control group. We found that learning spatial layouts through updated tactile feedback on programmable displays outperforms conventional procedures on static tactile maps. This could represent a powerful tool for navigation, both in rehabilitation and everyday life contexts, improving spatial abilities and promoting independent living for VI people.

## 1. Introduction

Navigating an unfamiliar environment is an ability that requires understanding of the spatial relationships between one’s self, proximal and distal objects [1]. The mental representation describing the surrounding space is also known as a spatial cognitive map [2,3]*.*

Spatial cognitive maps are usually built thanks to visual cues signaling the location of landmarks [4,5], although several studies indicate that blind people maintain the ability to represent spatial information, as well as to recognize a traveled route [6,7], thanks to the contribution of the other sensory modalities (mainly audition and touch). 

Nevertheless, other studies have reported difficulties for totally congenitally blind performing spatial cognition tasks (see for a review [8]). For instance, blind individuals might prefer route-like representations of spatial information while those who are sighted tend to code spatial information as survey-like representations [9,10]. A route representation is *egocentric* as it is based on the perspective of a ground-level observer. As a consequence, it is based on egocentric coordinates such as ‘left’, ‘right’, ‘ahead’, ‘behind’. On the contrary, a survey representation is defined as *allocentric* because it is based on an external perspective, independent from the observer [11]. Therefore, it is based on allocentric coordinates such as ‘North’, ‘South’, ‘East’, ‘West’. While both representations allow, in principle, successful navigation, survey knowledge is considered as more flexible since it permits the subject to mainly plan navigational paths, but also to take shortcuts or deviations from learned routes if unexpected obstacles appear [12,13,14,15]. Indeed, survey strategies are usually associated with higher spatial performance both in sighted [12,16] and blind subjects [17], even if they are harder to learn [18,19,20] but possible for blind persons [21,22,23,24]. The preference for route representations in the blind might be due to the fact that they correspond more to what is learned by experience: visually impaired individuals mainly acquire serial information while exploring the environment through touch, audition and motor information e.g., [25]. These contradictory findings might be due to task-dependent factors or related to different compensatory strategies as well as to the different mobility skills of the participants [6,9,17,26,27,28,29].

Tactile maps are useful tools in mobility training since they only include essential spatial information while providing a global representation of the relevant environment [30,31,32]. Several studies have investigated how visually impaired persons can take advantage of tactile maps in distance estimation [33,34] and self-location [35]. Tactile and audio-tactile maps have also been shown to be more useful than other methods such as verbal description [36,37] or direct experience with the environment [38], although the visually impaired may need some training to use them proficiently because scale factors are rarely given [34]. 

While traditional tactile maps, i.e., planar representations of spatial layouts with reliefs, such as those prepared using microcapsule or thermoform methods are still largely used, some recent technological advancements are able to overcome apparent limits of the traditional methods. For instance, pin array matrices (PAM) allow interactivity and real-time dynamic re-drawing of planar tactile maps through refreshable information [39]. There is also evidence showing higher user satisfaction when using interactive compared to traditional maps [40,41], studies that consider tangible interfaces [42] and low-resolution tactile displays [43,44] as possible substitutes for planar maps. Despite the evident utility of tactile maps, little studies have been done aiming at investigating their effectiveness as mobility aids in real environments [36,37,45,46] and few studies exist with regard to PAM [47,48,49,50,51]. 

However, none of the previous studies have assessed how blind persons, in orientation and mobility tasks, can use pin arrays to learn an offline tactile map beforehand and then to update their cognitive map and plan a different navigation path (performed without the PAM) based on an allocentric update of their position. Specifically, most of the previous studies treated PAM as obstacle avoidance or on-line travel planners, something which forces VI persons to carry the device and continuously get feedback; however, no study considers PAM as a dynamic tactile map to be studied before navigation and to be updated offline. In other words, instead of considering PAM as electronic travel aids (something that does not guarantee to efficiently build cognitive maps), here we propose to use them as electronic support to build and refine cognitive maps, while the navigation is a process entirely performed by the VI with no technological aids.

In our study, we aimed at investigating whether visually impaired individuals can understand and take advantage of tactile maps presented on a PAM to form a spatial cognitive map and to effectively reduce navigation errors in a real indoor environment. In particular, we wanted to find out whether a ‘touch the *updated* map, then re-explore’ strategy is associated with lower navigation errors when locating a target position in a real environment, rather than a ‘touch the *non-updated* map, then re-explore’ strategy, that simply offers a map than never changes and does not update tactile information. To do so, we presented a map on a PAM depicting an indoor environment and a position to be reached to two groups of visually impaired individuals. One group also received tactile feedback showing the hypothesized position previously reached while the other group could only explore the same map again. Another goal of this study was to find out whether visually impaired participants behave differently in adopting route vs. survey perspectives both in terms of map comprehension and navigation ability. To answer this question, we presented the tactile maps either in a more egocentric perspective (i.e., with the entrance door on the bottom of the map) or in a more allocentric perspective (i.e., with the entrance door on the top of the map). 

A final aim of the study has been to evaluate the level of understanding of tactile maps using two different methods to externalize a visually impaired knowledge [52,53]: a reconstruction of the map and a verbal questionnaire including both allocentric and egocentric features. We also verify how the scores of these externalization methods correlate with navigation performance.

## 2. Materials and Methods 

### 2.1. Participants

A group of blind (BLI) and low-vision (LOW) participants was recruited by Istituto Chiossone Onlus of Genoa. Participants were divided in an experimental (EXP) (*n* = 10; age range: 12–41 years; mean = 23.5; 5 blind; 4 females) and a control (CTR) (*n* = 8; age range: 13–28 years; mean = 17.5; 4 blind; 7 females) group. All participants were naïve to the experiment and none had a cognitive impairment that could influence performance in the tasks. The participants’ family gave informed consent in compliance with the Declaration of Helsinki. The experimental protocol was approved by the local Ethics Committees.

### 2.2. Programmable Tactile Display

**Figure 1:**
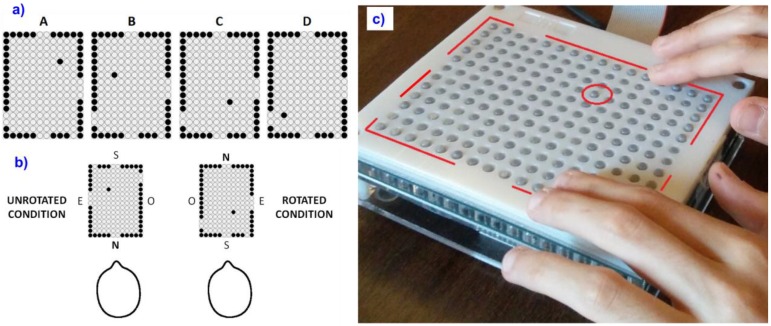
(**a**) The four maps used in the study with North shown on top. Note as the maps differ only for the virtual target location indicated by the single raised taxel inside the room; (**b**) Each map could be either shown in an unrotated condition (left) or in a rotated condition (right), depending on the North location, in counterbalanced order; (**c**) Map B displayed, unrotated, on BlindPAD, the pin array matrix we used in the study. The red lines highlight the walls and the target location.

The experiment was performed using a PAM named BlindPAD (Figure 1a). It is a refreshable multi-line tactile display composed of 192 moving pins (*taxels*, i.e., tactile equivalent of the pixels) on an 8 mm pitch [54]. Each taxel is individually programmable to be in the ‘up’ or ‘down’ state in under 20 ms and the whole matrix is refreshable in under 2 s, thanks to the 12 × 16 array of electromagnetic actuators and to the electronic control board. For the purpose of the study, the PAM was connected via wireless to a standard laptop and controlled by PadDraw, a software developed by Geomobile GmbH, Germany. The actuation principle and the hardware were designed and built at EPFL, Switzerland [54]. BlindPAD and PadDraw were developed within the scope of the FP7 EU BlindPAD project [55]. 

The BlindPAD display presents several technical novelties:

**A low-resolution tactile display**. The first novelty lies in the presentation of tactile graphics with a pin array that is not a Braille display. Braille-based displays have been shown to be effective in presenting maps to blind pedestrians [48] or in presenting simple geometrical dispositions to blind youngsters [39]. In the literature, a modified disposition of Braille dots is able to convey graphics, where the spacing between dots is constant (i.e., 2.5 mm, see Table 1). Instead, Braille dots that convey textual information do not require constant spacing (i.e., 2.5 mm within the same character and 3.5 mm between adjacent characters). Therefore, prior research shows that Braille-based dot spacing is sufficient to perceive and understand graphical information.

However, there is a lack of clarity on whether or not Braille specifications are also necessary. Specifically, does a simple sketch of a room require a Braille resolution? If this is the case, then decreasing the resolution would result in unclear understanding of space.

Here, we reason that if one decreases the resolution of a pin array tactile display, each dot (a taxel) becomes larger, and so it goes for the dot spacing. However, keeping approximately constant the diameter/spacing ratio allows to maintain the proportions between the information in relief (i.e., the taxels) and the flat background surrounding the taxels. In practice, we built a ‘zoomed’ version of a Braille graphical display. The spacings of the BlindPAD are very similar to those of the standard LEGO dots (see Table 1) that were used in our experiments, which have already shown to help with the construction of tactile maps [56].

We hypothesize that learning spatial layouts with a resolution lower than Braille does not impair their understanding. We further hypothesize that a single dot can convey information on the exact location of a target.

**A sufficiently high blocking force.** Each taxel is magnetically actuated [54]. One unique feature of this device is that, unlike piezoelectric Braille bars, it has a lower latching force, i.e., the blocking force that allows a taxel to hold its ‘up’ position before being cast by the finger at the same level of the flat background. While piezoelectric Braille dots can stand forces of several Newtons, here we assume that such a high blocking force, although sufficient, is *not* necessary. This is confirmed by previous studies demonstrating that perception threshold of forces on a single dot are as low as 40 mN for dots having a similar stroke than BlindPAD [58]. Our dots have a latching force of 200 mN [59], i.e., five times the perceptual threshold, therefore large enough to be perceived by end users but much smaller than that of standard piezoelectric-based dots.

**An ergonomic form factor.** BlindPAD is a non-square tactile graphical screen. Its 12 × 16 taxels allow to cover an area of 94 mm × 124 mm. These measures were chosen for two reasons: to allow the perception of all taxels on the longer side of 124 mm with both hands (the digits 2, 3, 4, 5 of both hands of an adult resting side-by-side on the device would cover an average breadth of 124 mm for males and 108 mm for females) and to maintain an aspect ratio of 4/3, i.e., common to visual graphics [60].

### 2.3. Stimuli

Four maps of a room were prepared on the BlindPAD (see Figure 1a). The maps depicted the essential features of the room (i.e., walls, doors) complying with the dimensions of the real room (4.5 m × 6 m, see Appendix A). The maps also included a cue (i.e., a single taxel raised up) which indicated a virtual target position that participants had to reach (see Figure 1a). We named the target as ‘virtual’ because it simply indicated a position to be reached without an actual physical object placed at that location in the room. Since the four maps represented the same room, they only differed in the position of the virtual target. Tactile maps were concurrently presented to the participants with a synthetized audio description of the room providing also cardinal directions (e.g., “You will be entering the room from the door placed on the North side of the room”). See Appendix A for a complete description of the auditory information provided to the participants.

### 2.4. Map Reconstruction

**Figure A1:**
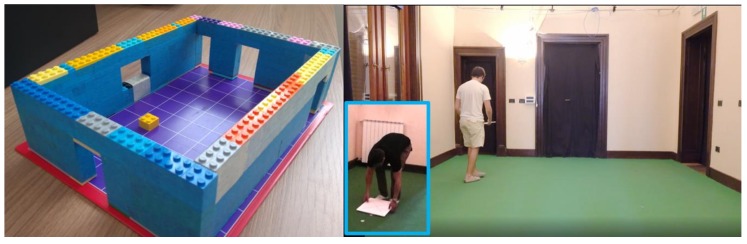
**Left**: Example of a map reconstructed with LEGO bricks. The four walls were presented detached from the magnetic floor. **Right**: Participant performing the navigation task.

LEGO (Billund, Denmark) bricks were used to prepare a set of modules that participants used to build a 3D model of the room after tactile map exploration (see Figure A1 in Appendix A). Each module represented a single wall of the room with its unique features (e.g., a door close to a corner). We attached small magnets to the bottom of each element to allow participants to place the wall on top of a magnetic surface easily. Note that the reconstruction task is a simplified version of a traditional reconstruction using simple, not assembled, LEGO bricks, as we prepared the main modules beforehand. The target (a small 2 × 2 LEGO brick) was also provided to the participants (see Figure A1). 

### 2.5. Map Comprehension Questionnaire (MCQ)

We also prepared a Map Comprehension Questionnaire (MCQ) to evaluate how visually impaired participants understood the tactile maps (see Table 2). The MCQ included three items (Items 1–3) assessing a route representation of the room (e.g., “which direction will you take to reach the target location? (e.g., left, right, straight ahead)”) and three items (Items 4–6) assessing a survey representation of the same room (e.g., “where is the window located? (e.g., North, South, East, etc.)”). Items 1 and 2 could be answered relying on verbatim memory alone, whereas Items 3–6 required the participants to make spatial inferences.

### 2.6. Procedure

Participants with some residual sight were blindfolded to avoid visual inspection of the materials. After that, they familiarized themselves with the tactile display and with the LEGO elements. 

The study comprised four sessions, one for each of the four maps (see Figure 1a), presented in counterbalanced order. Each session comprised three phases: (1) tactile map exploration; (2) multimodal map externalization; (3) physical navigation and review. In the tactile map exploration phase, the participants haptically explored one of the maps shown on BlindPAD while listening concurrently to the synthetized description of the room. Two maps were presented with the North on top (rotated condition, see Figure 1b) and two were presented with the North on bottom (unrotated condition), in counterbalanced order. In the multimodal map externalization phase, the participants exhibited their level of understanding of the cognitive map by two means. The first externalization mean was a reconstruction task: the participants were asked to build a 3D model of the map (see Appendix A), to match what they had previously perceived on BlindPAD. The second mean was an MCQ. No feedback on the performance was given to the participants at this stage. Finally, and immediately after, the participants performed the third phase: they physically navigated the real room, by always entering from the North-side wall. The participants were instructed to reach the hypothesized target position and to put a Styrofoam panel on the floor, i.e., where they thought the target location was. The experimenter measured the Euclidean distance between the correct target location and the hypothesized location—indicated by the participant—with a measuring tape. The participants had three attempts (trials) to reach the target. The kind of information received in-between these three attempts differentiated an experimental group and a control group.

**Figure 2:**
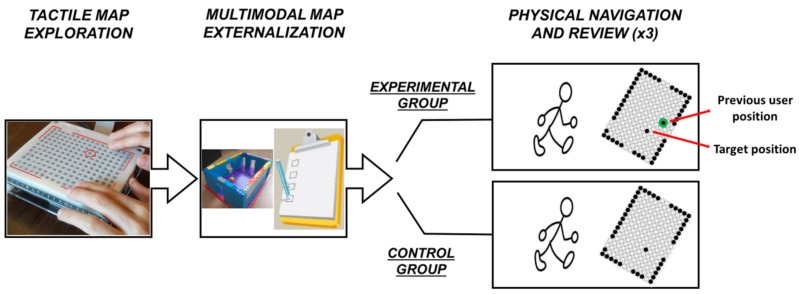
Procedure of a single session: both groups explored the map on BlindPAD, then externalized the map with LEGO modules and with a questionnaire; then, both groups navigated in the real room three times. The experimental group received feedback about the hypothesized (in green) and the target position, while the control group only reviewed the initial map. Each participant sustained four sessions: what varied was the displayed map, i.e., the different target positions as in Figure 1a.

The experimental group obtained feedback on BlindPAD (one raised taxel, see Figure 2) about the previously hypothesized target location, together with the actual target location (another raised taxel). In order to avoid possible confusion between the actual and hypothesized target location, we always presented the target first, followed by the position reached. The goal was to help the implicit planning of the next physical navigation, based on the explicit representation of the past reached position. The feedback on BlindPAD about the hypothesized position was given three times. Therefore, the participants navigated three times for each session. Whenever the participant reached the real target location, only the target position was shown in the next map presentation.

However, the control group only re-explored the same map before each navigation: the reviewed map only contained the target position. The goal was to account for learning effects only.

Each session lasted about 20 min, for a total duration per participant of about 90 min and a total data collection of 30 h. All the experimental sessions were videotaped.

### 2.7. Statistical Analyses

Concerning the independent variables, we defined the kind of feedback (i.e., updated vs non-updated map), the visual disability and the rotated/unrotated condition of the map as between-group factors, while the session and the trial were within-group factors.

Concerning the dependent variables, we assessed the second experimental phase, i.e., the multimodal map externalization. In particular, as for the reconstruction task, we considered the accuracy as well as the time spent by the participant to complete the reconstruction. For the accuracy, we assigned a score = 100% if the participant placed all the 5 modules (4 walls and 1 target) correctly. For each wall, there were three possible scores: 0 = wrong selection, 50% = right wall but flipped, 100% = correct. For the target, only two scores were possible: 0 = wrong position, 100% = correct. The target was placed correctly if the participant guessed the correct among four quadrants (i.e., Northeast, Northwest, Southeast, Southwest) in which it was located. Hence, each reconstruction was assigned a mean accuracy score depending on the average of the sub-scores described above. 

As for the MCQ, 5 out of 6 items were assigned a score of 1 in case of a correct response and a score of 0 in case of a wrong response. Item 6 required the participants to estimate the distance of the target from the entrance. Hence, for this item we analyzed a measure of error in meters.

We also assessed the third experimental phase, i.e., the physical navigation task. In particular, we measured the error in meters as Euclidean distance (i.e., distance between the target and the Styrofoam panel) as well as the time to place the panel since the beginning of the trial.

Since data were not normally distributed (Shapiro–Wilk test), we employed non-parametric statistics. In particular, within-group statistics were performed either with Wilcoxon signed-rank or Friedman ANOVAs tests. Between-groups statistics were performed using Mann–Whitney U tests. 

In addition, Spearman correlation tests were run to investigate relationships between variables.

Correction for multiple comparisons, whenever needed, was conducted using the False Discovery Rate (FDR) control based on the Benjamini–Hochberg methods [61,62]. 

## 3. Results

In the following subsections, we present the results of the multimodal map externalization and the physical navigation, as well as their correlations.

### 3.1. Multimodal Map Externalization

#### 3.1.1. Map Reconstruction

The average accuracy of the participants in reconstructing the 3D model with LEGO modules was 64% with a completion time of 119 s. We could not find any difference related to the level of visual disability, session, condition, or target position (all *p*s > 0.05). 

An inverse correlation between accuracy and reconstruction time emerged both in BLI and LOW (*r* = −0.49 and *r* = −0.45, respectively, both *p*s = 0.006). 

#### 3.1.2. Map Comprehension Questionnaire

**Figure 3:**
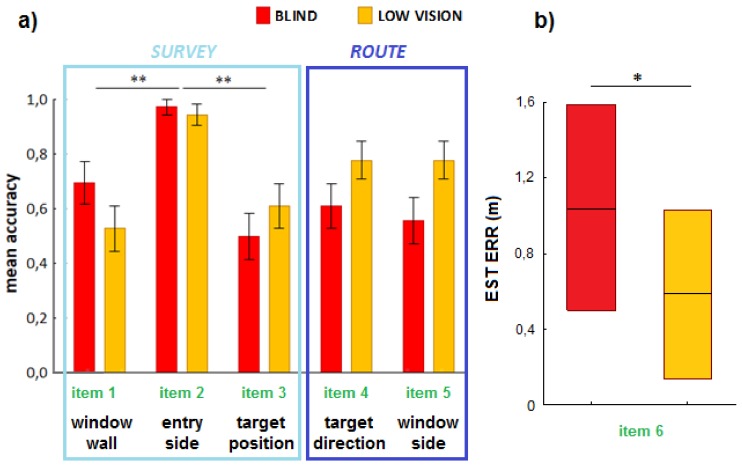
(**a**) Accuracy scores for each item of the MCQ by level of visual disability. Asterisks indicate higher accuracy for Item 2 than Items 1 and 3, both for blind (BLI) and low-vision (LOW) participants. **, *p* < 0.01; (**b**) Estimation error (m) relative to the distance to reach the target location. Asterisk indicates a significantly larger estimation error in the BLI compared to LOW participants. *, *p* < 0.05.

Friedman ANOVAs indicated differences in accuracy between items in both groups (*χ^2^* = 19.9; *p* = 0.00005). Accuracy for Item 2 (entrance side) was indeed higher than accuracy for Items 1 and 3, which were both in the BLI and LOW (all *p*s < 0.05; see Figure 3a). BLI and LOW only differed for the accuracy of Item 6. LOW were indeed more accurate than BLI in estimating the distance to walk (*U* = 404.5, *p* = 0.04; see Figure 3b).

We also checked whether this estimation error was differently related to each of the four target positions (see Figure 1a). This effect was especially evident for target position C for which the estimation error in the BLI was higher than in the LOW (*U* = 10, *p*FDR-corrected = 0.04).

We could not find neither a session nor a condition effect (all *p*s > 0.09).

#### 3.1.3. Correlation between Map Reconstruction and MCQ

We investigated whether the accuracy in building the model with LEGO modules and the reconstruction time possibly correlated with the scores of the MCQ and we assessed its possible dependence on visual disability. We observed significant correlations only for route items. In BLI, reconstruction accuracy tended to be negatively correlated with Item 6 (*r* = −0.39, *p* uncorrected = 0.017, *p*FDR-corrected = 0.1). We observed a negative correlation between map reconstruction time and Item 5 (*r* = −0.47, *p* uncorrected = 0.007, *p*FDR-corrected = 0.02) and a positive correlation between map reconstruction time and Item 6 (*r* = 0.46, *p* uncorrected = 0.006, *p*FDR-corrected = 0.02). 

In the LOW, unlike with BLI, we observed a positive correlation between reconstruction accuracy and route with Item 5 (*r* = 0.50, *p* uncorrected = .001, *p*FDR-corrected = 0.01) and Item 4 (*r* = 0.37, *p* uncorrected = 0.02, *p*FDR-corrected = 0.07). Similarly with BLI, in LOW LEGO accuracy also correlated negatively with Item 6 (*r* = −0.34, *p* uncorrected = 0.04, *p*FDR-corrected = 0.08). Finally, and again similarly with BLI, map reconstruction time tended to be negatively correlated with Item 5 (*r* = −0.33, *p* uncorrected = 0.046, *p*FDR-corrected = 0.27). All the other correlations were not significant (all *p*s uncorrected > 0.05).

Interestingly, the accuracy in positioning the target with LEGO was significantly higher than the accuracy in answering to the corresponding Item 3 of the MCQ (76% vs. 56%, *p* = 0.03), suggesting that the map reconstruction task might be a better solution than a verbal questionnaire to externalize visually impaired knowledge about a spatial map. 

### 3.2. Physical Navigation and Review Task

#### 3.2.1. Self-Positioning Errors in Navigation

When comparing the effect of an updated vs non-updated review, we first verified that the experimental (EXP) and control (CTR) groups did not differ in terms of errors at the baseline (1st trial of the 1st session). Indeed, we could not find any difference between groups in the Euclidean error in the first trial (*U* = 25, *p* = 0.19).

**Figure 4:**
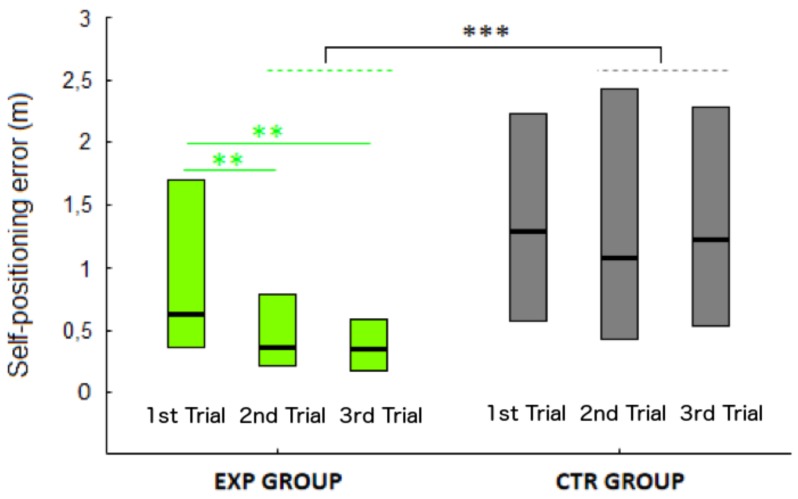
Self-positioning error (m) for each trial in the feedback group (green bars) and in the no-feedback group (grey bars). Green asterisks indicate a significant reduction of the self-positioning error in the 2nd and 3rd trial in the experimental (EXP) group. Grey asterisks indicate a significantly larger self-positioning error in the 2nd and 3rd trial in the control (CTR) compared to the EXP group. **, *p* < 0.01; ***, *p* < 0.001.

On the contrary, in the 2nd and 3rd trial (i.e., after reviewing the map) a clear effect of the updated presentation on self-positioning error emerged (see Figure 4). EXP error was indeed significantly lower than CTR error [*U* = 1123, *p* < 0.01]. This effect was evident regardless of the level of visual disability, condition, session and target position.

Therefore, the global self-positioning error, expressed as Euclidean distance from the hypothesized target location to the actual target location, was lower in the group that received an updated feedback while reviewing the map.

Beyond that, Friedman ANOVAs performed separately for EXP and CTR groups revealed an effect of trial only in the EXP (χ^2^ = 13, *p* = 0.002; see Figure 3) and not in the CTR group (χ^2^ = 1.71, *p* = 0.42). Euclidean errors in the 2nd and 3rd trial of the EXP were indeed significantly lower than in the 1st trial (both *p*s < 0.01). Therefore, there was a significant reduction of the self-positioning error trial-by-trial that was present only when the map was updated and absent when simply reviewing the non-updated map. 

We also verified whether self-positioning error was modulated by the session in the whole sample of participants. Euclidean distance indeed changed while proceeding with the sessions (χ^2^ = 11.67, *p* = 0.008). The error in the 4th session was lower than the errors in the 1st and 3rd session (both *p*s FRD-corrected < 0.05). This learning effect seemed to be especially evident in the BLI (χ^2^ = 10.73, *p* = 0.01) and absent in the LOW (χ^2^ = 4.53, *p* = 0.21).

As for the level of visual disability, in general, performance of BLI and LOW was comparable except for target position C (see Figure 1a), in which the Euclidean error was higher in the BLI (*U* = 212, *p* = 0.03).

Importantly, when presenting rotated maps with the North upside-down, the Euclidean error was higher than in the corresponding unrotated conditions both in the BLI and in the LOW (both *p*s < 0.05), suggesting there is a behavioral cost when forcing a VI to adopt a survey perspective.

#### 3.2.2. Navigation Time

**Figure 5:**
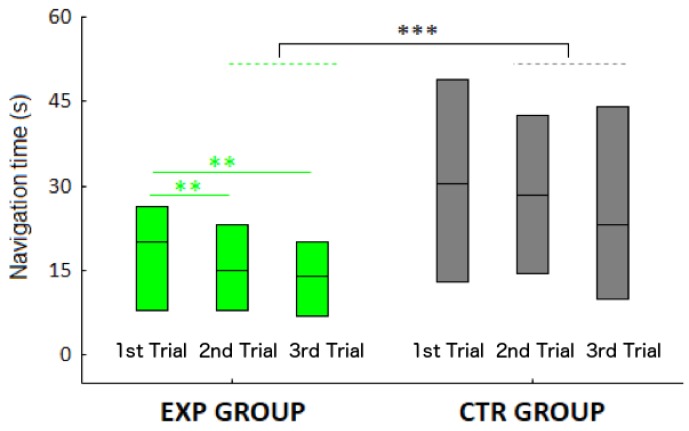
Navigation time (s) for each trial in the feedback group (green bars) and in the no-feedback group (grey bars). Green asterisks indicate a significant reduction of the navigation time in the 2nd and 3rd trial in the EXP group. Grey asterisks indicate a significantly slower navigation time in the 2nd and 3rd trial in the CTR compared to the EXP group. **, *p* < 0.01; ***, *p* < 0.001.

Navigation time data substantially confirms what we have seen with accuracy data (see Figure 5). Additionally, in this case we could not find a difference between EXP and CTR in navigation time of the 1st trial of the 1st session (*p* = 0.23). 

On the contrary, EXP participants were significantly faster than CTR when considering the 2nd and 3rd trials (*U* = 1628, *p* < 0.001). Therefore, the updated map led the participants to be faster than those who did not receive an updated map. However, when considering the level of visual impairment, the effect of an updated map was evident only in the BLI (*U* = 295, *p* < 0.001). 

When considering the session, the effect of an updated map only emerged in the 1st session.

Friedman ANOVAs performed separately for EXP and CTR groups revealed an effect of trial only in the EXP (χ^2^ = 11.05, *p* = 0.004; see Figure 5) and not in the CTR group (χ^2^ = 1,28, *p* = 0.53). Navigation times in the 2nd and 3rd trials of the EXP were indeed significantly faster than in the 1st trial (both *p*s = 0.009). Therefore, an updated map significantly reduces the completion time trial-by-trial.

As for the effect of the session, we could not observe significant differences in navigation time across sessions (χ^2^ = 3.47, *p* = 0.32). 

As for the level of visual disability, BLI were faster than LOW (*U* = 4877, *p* = 0.048).

As in the case of self-positioning errors, navigation times were slower in the rotated condition (*p* < 0.001).

Finally, a significant weak correlation between Euclidean error and navigation time emerged (*r* = 0.22, *p* = 0.001).

#### 3.2.3. Correlations of Self-Positioning Errors with Measures of Externalization 

We sought for possible correlations between the level of understanding of a map, expressed by the analysis on externalization techniques, and the actual performance in the real environment, expressed by self-positioning errors. We found that both reconstruction accuracy and reconstruction time were significant predictors of performance in navigation: in fact, the self-positioning error in navigation inversely correlated with LEGO accuracy (*r* = −0.43, *p*FDR-corrected < 0.001) and directly with reconstruction time (*r* = 0.52, *p*FDR-corrected < 0.001). 

Interestingly, the navigation time directly correlated with map reconstruction time (*r* = 0.42, *p*FDR-corrected < 0.001).

As for the correlation between navigation task and MCQ, we could observe significant correlations only with the route items. Particularly, the self-positioning error negatively correlated with Items 4 and 5 (*r* = −0.45 and *r* = −0.35, respectively, both *p*s FRD-corrected < 0.001) and positively with Item 6 (*r* = 0.36, *p*FDR-corrected < 0.001).

Overall, the map reconstruction seems to be a better way to externalize map knowledge in the VI. Both the considered variables of the reconstruction task (i.e., accuracy and time) moderately correlated with the considered navigation task variables. As for the MCQ, only the route items were predictive of the accuracy of the navigation task. 

## 4. Discussion

The main aim of this study was to evaluate the extent to which tactile feedback reduces self-location errors in navigational tasks involving visually impaired people. To do so, we presented to visually impaired participants a tactile map with a pin array matrix that displayed an indoor environment. The map also included a position to be reached inside the room. After the first physical navigation, one group also received tactile feedback showing the position they actually reached, whereas a control group only reviewed the tactile map without the feedback cue. This is a novel approach because most previous studies assessed the role of electronic devices as ‘obstacle detectors’ or, more in general, as wayfinding aids to be carried by the user e.g., [63,64]. Instead, we propose to provide feedback as a tool to explicitly update the spatial cognitive map of the user and implicitly support planning of different navigation paths (without the pin array matrix) based on an allocentric update of his/her position. In other words, we seek to use sensory feedback to reinforce the construction of a cognitive map prior to navigation, instead of giving information about the environment while navigating.

We observed that providing tactile feedback allowed visually impaired participants to considerably reduce the mean localization error of the target and the mean time required to complete the task already in the second trial (from 106 to 59 cm and from 21 to 18 s). On the contrary, the performance of the control group which did not receive the feedback did not change significantly between trials.

The performance improvement originated by an updated map was evident regardless of the level of visual disability, suggesting that totally and congenitally blind individuals can also take advantage of allocentric information, as shown by previous studies e.g., [21,22].

Arguably, our results suggest also that technological aids for navigation of blind persons do not need to be introduced necessarily *while* navigating, but they are effective also *prior* to navigation. In particular, the absence of a significant reduction in self-positioning errors in the control group indicates that experience alone—which leads to learning by repetition—may be a weaker improvement method compared to the benefit of the updated information originated by technological means. Had we observed the control group only, we could not have inferred whether the amount of self-positioning error was possibly due to misunderstanding of the map, because overall the self-location error did not change after three trials even if our participants did review the non-updated map. One of the problems to solve is how to eliminate the sense of frustration when one thinks one has mentally well-constructed or understood a map and then realizes that, after a physical navigation, the target is elsewhere. A similar sense of frustration affects rehabilitation practitioners, since the cause of the misunderstanding is frequently unknown. This is in line with current rehabilitation practices, in which it is known how incredibly long physically learning a spatial layout takes, and how to relate that layout to a map e.g., [65]. Instead, observing a reduction in self-location errors in the experimental group clearly indicates the usefulness of an a priori allocentric map, because self-location errors significantly reduce immediately after the first trial. We think that the representation of prior performance with touch provides a lot of useful information: 1) it confirms whether anchor points are located where the user thinks they are; 2) it implicitly gives a scale factor, because the walked distance can be related to the space that still has to be covered to reach the target; 3) it allows the user to relate the hypothesized target position with other elements of the map: we speculate that adding a single tactile element to the map has proved to be so efficient because the erroneous target positions become a novel reference point of the map from which the participant can make further spatial inferences.

Importantly, the dynamic presentation of maps can also be a way *per se* to verify if a map is understood or not. Varying the tactile feedback on a map can therefore be a powerful tool in rehabilitation contexts, which sometimes lack assessment means. It can also considerably steepen learning curves and help blind persons to autonomously verify the amount of self-location errors in unknown environments, with evident implications for independent living. For instance, we can imagine a real-life scenario in which a supermarket is provided with a tactile screen on each shelf signaling to the visually impaired persons the location of each kind of goods (e.g., vegetables, cheeses). When touching the map, the blind person would be able to estimate the distance from the desired shelf. 

The amount of reduction in self-location errors is higher than the precision of the BlindPAD device in terms of tactile feedback, therefore the resolution of the pin array matrix is largely sufficient for this task. Specifically, a 6 m × 4.5 m room was represented on a 12 × 16 pin array matrix, therefore each taxel represented a self-positioning error square (i.e., an equivalent real spatial resolution) of about 0.35 m (see Appendix A), i.e., much lower than a typical walking stride length of 0.8 m. Clearly, the question whether resolution or not is a determining factor is task-specific: using BlindPAD outdoor could be equally sufficient if self-positioning errors increase at the level of meters (which appears reasonable).

On the other hand, our results also highlight a behavioral cost when asking a visually impaired person to use an allocentric representation. Both navigation accuracy and speed decreased when presenting a rotated map. This happened since the participant had to make an extra effort to adopt an egocentric representation, because the entrance door (always to the North) was presented on top of the map. However, these effects were present not only in blind but also in low-vision participants suggesting that they might not be an effect of visual disability per se but rather an effect of switching to a more complex spatial ability [25] which might even involve different neural circuits e.g., [66]. This also implies that North-sided maps, which are a convention on printed papers, may no longer be a requirement on dynamically presented maps on tactile devices. Similarly to the egocentric navigational cues that drivers obtain on flat screens of their dashboards, visually impaired persons can benefit from auto-rotated maps that are still top-view, but at least align the direction of walking to the body midline, therefore limiting the number of mental rotations, which are known to be cognitively costly [67,68].

Although the sample sizes of this study did not allow us to make strong inferences about possible differences/similarities between blind and low-vision participants, in general, we observed that the performance of the two groups were comparable except for a specific position of target (Map C) for which localization errors were higher in blind participants. These errors were predicted by the higher error in verbal distance estimation. Map C looks special in that the target is further away from the walls as compared to any other map. The lack of close reference points (i.e., landmarks) such as the walls might explain the result e.g., [69]. This is supported by a significant positive correlation (*r* = 0.2, *p* = 0.04), only in the blind, between self-positioning error in the navigation and the distance between the target and the nearest wall.

Another aim of this study was to evaluate the use of two different externalization methods to evaluate how much visually impaired participants understand tactile maps. To do so, after the exploration of the tactile maps, the participants first performed a map reconstruction using LEGO bricks assembled as modules, then completed a verbal questionnaire composed of both egocentric and allocentric items. Although there is a considerable amount of literature on externalization methods in the blind [52,70], less is known when the externalization follows the exploration of maps presented with pin array matrices [53] and no studies have attempted to correlate the externalization scores with the actual navigation performance. In our study, visually impaired participants obtained a similar accuracy when externalizing the map via reconstruction and verbal questionnaire. The mean accuracy with the reconstruction method was 64% against 70% with the first five items of the verbal questionnaire. However, the verbal questionnaire included an item (Item 2: “Which side hosts the entrance?”) in which the mean accuracy was extremely high (96%) probably because the answer was fixed across maps and participants could rely on verbatim memory alone. The mean score in the verbal questionnaire excluding Item 2 was 63%, i.e., almost identical to the reconstruction score. Nevertheless, some differences between the two methods emerge when considering their correlations as well as the correlation with the actual navigation performance. For instance, the accuracy in positioning the target with the LEGO (76%) was higher than the accuracy of Item 3 (56%) that required participants to report where the target was located in allocentric coordinates (e.g., Southeast). This dissociation in performance suggests that some visually impaired participants might have had difficulties in using cardinal coordinates in the verbal externalization. In addition, both map reconstruction accuracy and time correlated moderately with navigation performance whereas only the route items of the verbal questionnaire correlated with navigation accuracy. Indeed, the route-items were required to make spatial inferences and could not be answered based on verbatim memory alone. Hence, when using verbal questionnaires, a careful selection of the items seems to be advisable. The items requiring spatial inferences might be a more reliable way to assess the understanding of spatial tactile maps. 

One limitation of this study is the small sample size which makes it difficult to draw solid conclusions about possible differences or similarities between blind and low-vision participants. Furthermore, we deliberately opted for a simple testing environment (a small empty room) because we aimed at investigating the pure role of tactile feedback administered in the simplest situation before investigating more complex environments. 

Future studies might wish to reproduce our results with larger sample sizes, more complex environments and/or outdoor testing.

## 5. Conclusions

In this work, we showed that providing tactile feedback about self-position on a map displayed with a pin array matrix improves the orientation abilities of visually impaired persons better than reviewing the non-updated map. We also showed that for visually impaired persons a model reconstruction of the map might be a better externalization method than a verbal questionnaire.

## Figures and Tables

**Table 1:** Physical characteristics of dots in three different technologies: BlindPAD shares a similar resolution to LEGO dots, but with a stroke similar to Braille dots and a diameter/spacing ratio common to both technologies.

PAM Characteristics	Standard Braille [57]	BlindPAD	LEGO
Dot diameter	1.52 mm	4 mm	4.8 mm
Dot spacing (center-to-center)	2.54 mm	8 mm	8 mm
Stroke (displacement)	0.6 mm	0.55 mm	1.8 mm
Diameter/spacing ratio	0.6	0.5	0.6

**Table 2:** Map comprehension questionnaire. Items 1–3 are survey items because they required an allocentric answer (e.g., North, East, South-West, etc.). Items 4–6 are route items because they required an egocentric answer (e.g., right, left, ahead, etc.).

Map Comprehension Questionnaire (MCQ)		
	Item 1	Which side has the window?
Survey items	Item 2	Which side hosts the entrance?
	Item 3	Where is the virtual target?
	Item 4	Which direction you should walk to reach the target?
Route items	Item 5	On which side you will find the window from the entrance?
	Item 6	How distant is the virtual target from the entrance? (in meters)

## Appendix A. Map Reconstruction Task and Room Description

In the map reconstruction task (see Figure A1, left) the participants were presented with the four walls of the room in a pile, with no specific order, and a small piece representing the target. The single walls were already assembled, and the bottom part of each wall was taped with a magnetic stripe. The floor on which the walls had to be placed was magnetic, to minimize unintentional motion of the walls. Possible sources of mistake were the placement of the walls in the correct cardinal position and the way the wall was flipped (neither wall was symmetrical with respect to wall entrances).

The real room was 6 m × 4.5 m, and it was completely empty (see Figure A1, right) and covered by a thin carpet to minimize acoustic cues from the floor. It had four entrances and one window, all of them represented on both the BlindPAD and the LEGO walls, with the precision allowed by each technology. The precision of a single taxel of BlindPAD, projected in the real room, was a rectangle of 0.33 m × 0.37 m and corresponded to the dimensions of the Styrofoam panel. This indicated an explicit physical scale factor to the participant. Figure A1 shows one participant walking through the room and another participant (blue-framed subfigure) placing the panel on the floor as instructed.

The text of the audio synthesized voice that described the room is reported below:

- Good morning. I will now describe the room. The room has a rectangular shape. It is 6 m × 4.5 m. Inside there are three big wooden doors, a small door and a wide window. The three doors and the small door are covered by fabric.
- [Rotated condition] The North side of the map lies at the top of the device. [Alternatively, Unrotated condition] The North side of the map lies at the bottom of the device.
- On a 4.5 m long side, to the North, there is a big wooden door. On a 6 m long side, to the East, there is a wide window. On a 4.5 m long side, to the South, there is a big wooden door. On the same side, in the Southeast corner, there is a small door. On a 6 m long side, to the West, in the Southwest corner, there is a big wooden door.
- Inside the room there is a square. The position of the square indicates where you will have to place the object. You will enter through the North side.
